# New Frontiers in Myopia Progression in Children

**DOI:** 10.3390/jcm13237314

**Published:** 2024-12-02

**Authors:** António Queirós

**Affiliations:** 1 Clinical and Experimental Optometry Research Lab (CEORLab), School of Science, University of Minho, 4710-057 Braga, Portugal; aqp@fisica.uminho.pt; 2 Physics Center of Minho and Porto Universities, 4710-057 Braga, Portugal

## 1. Introduction

Myopia is a growing public health issue, with projections indicating that half of the global population may be affected by 2050 [1]. In recent years, the increasing incidence of myopia in children has drawn the attention of researchers and healthcare professionals worldwide. This condition extends beyond mere difficulty in seeing distant objects; it is associated with an elevated risk of severe ocular complications, including retinal detachment, early cataract, and glaucoma, particularly in cases of higher myopia [2]. These complications can lead to permanent vision loss and are expected to have a significant socio-economic impact, particularly on the healthcare systems of various countries [3].

The risk factors for the development and progression of myopia are complex and multifactorial, involving both genetic and environmental aspects. Reduced exposure to natural light, prolonged time spent on near activities such as reading and using digital devices, and familial predisposition are some of the well-documented factors contributing to the rising prevalence of myopia in children. Recent studies have underscored the importance of understanding the interplay among these factors to develop effective interventions [4].

In this context, the Special Issue, “New Frontiers in Myopia Progression in Children”, aims to highlight the latest advancements in the understanding, prevention, and treatment of myopia progression. Among the 27 studies presented, original research articles offer new insights into the impact of various interventions, such as the use of myopia control contact lenses, orthokeratology lenses, and low-dose atropine administration. Furthermore, review articles provide a comprehensive synthesis of recent findings, pointing to future directions in research and clinical practice.

In this issue of the *Journal of Clinical Medicine*, we highlight a total of 27 articles focused on the advances and challenges in myopia progression control. Of these, 22 are original research studies involving 6886 subjects with a mean age of 14.5 ± 6.8 years, providing a solid empirical evidence base. The remaining five articles are reviews that synthesize current knowledge and provide a critical analysis of best practices and existing gaps in the literature. The collaborative and global nature of this edition is noteworthy, with contributions from 144 authors across 12 countries, reflecting the universality of the issue and international efforts to find solutions. The studies compiled in this edition feature 130 keywords, among which the most frequent are “myopia” (13 occurrences), “myopia control” (10 occurrences), and “refractive error” (8 occurrences). Other terms such as “orthokeratology” (6 occurrences), “atropine” (4 occurrences), and “axial length” (4 occurrences) indicate the commonly investigated treatment approaches and measurement parameters. This collection of articles provides a comprehensive overview of associated risk factors, such as genetic and environmental interaction, as well as strategies for controlling progression and the challenges in developing effective therapies.

The research and technological advancements discussed in this Special Issue are crucial for shaping more effective prevention and intervention strategies. From the use of specialized lenses that alter peripheral refraction to pharmacological approaches such as low-dose atropine, recent developments aim not only to slow the progression of myopia but also to enhance the quality of life for young patients. However, challenges persist, including the need for long-term studies and the adaptation of treatment strategies for different populations and cultural contexts.

## 2. Epidemiological and Genetic Factors

Epidemiological studies have highlighted a significant occurrence of pre-myopia in children, underlining the importance of early interventions to prevent progression. Additionally, genetic factors play a crucial role in the development of myopia, with a family history of the condition notably increasing the risk. Research also suggests that specific genetic variations can influence how children respond to treatment, pointing towards the potential benefits of personalized approaches for managing myopia. These findings reinforce the idea that myopia management should consider both environmental and genetic factors for more effective, individualized care [5,6].

Modrzejewska and Durajczyk investigated 1155 eight-year-old children in Poland and found that pre-myopia was diagnosed in approximately 60.9% of participants, highlighting the importance of early interventions to prevent progression to significant myopia (Contribution 1).

Similarly, Okabe et al. demonstrated in their analysis of eight-year-old Japanese children that heritability directly influences myopia development. Children whose parents had a history of glasses usage exhibited a significantly higher risk of developing myopia (Contribution 2).

Genetic studies, such as that by Alvarez-Peregrina et al., complement these findings by exploring how specific genetic variants may modulate treatment response. In particular, this study showed that certain genetic variations were associated with better responses to myopia control lens treatments, suggesting potential for personalized treatment approaches based on the patient’s genetic profile (Contribution 3).

## 3. Optical and Pharmacological Interventions

Advancements in optical and pharmacological treatments have played a crucial role in controlling myopia progression. Optical interventions, such as orthokeratology (ortho-k) and multifocal contact lenses, help slow axial elongation by reshaping the cornea and reducing peripheral hyperopia. On the pharmacological side, low-dose atropine has been shown to effectively slow myopia progression, especially in children, with minimal side effects. These combined approaches offer a promising strategy to manage myopia and reduce long-term risks [7,8,9,10].

Amorim-de-Sousa et al. conducted a pioneering study assessing the impact of radial gradient power contact lenses on choroidal thickening and retinal electrical response. Results indicated that these lenses might induce an increase in choroidal thickness and alter the electrical response of the retina, suggesting possible mechanisms for controlling myopia progression (Contribution 4).

Orthokeratology has also been extensively studied as a method for controlling myopia progression. Loertscher et al. conducted a paired-eye study comparing multifocal orthokeratology lenses with conventional lenses. The results showed that multifocal lenses significantly reduced axial growth, pointing to their superior efficacy over traditional lenses (Contribution 5).

Similarly, Pauné et al. investigated how the diameter of the posterior optical zone of orthokeratology lenses may influence myopia progression, revealing that smaller diameters induced less axial growth and, therefore, were more effective. The authors present a summary table relating the optical zones of the lens to the pupil diameter of the subjects (Contribution 6).

According to Silva-Leite et al., perifocal ophthalmic lenses have shown promise in controlling myopia progression by inducing specific peripheral defocus patterns that may enhance visual function. Their research indicates that these lenses create a myopic defocus in the nasal retina, which is crucial for managing eye growth and potentially reducing myopia progression (Contribution 7).

Low-dose atropine has emerged as a prominent pharmacological treatment, with studies confirming its efficacy in slowing myopia progression. Myles et al. reported that administering 0.01% atropine to Australian children significantly reduced the rate of myopia progression with minimal side effects and suggested that treatment should commence as early as possible in at-risk children (Contribution 8).

In a meta-analysis conducted by Lee et al., it was found that low-dose atropine was not only effective in reducing the incidence of myopia in pre-myopic children but also controlled progression over 12 to 24 months without major adverse effects (Contribution 9).

## 4. Psychological Impact and Quality of Life

While the primary focus of interventions for myopia control is clinical efficacy, the impact on children’s quality of life is also a vital aspect [11,12].

Michalski et al. explored how low-dose atropine use affects the psychological well-being of adolescents. The study revealed that although there were some complaints regarding near activities and glare, participants reported a high level of confidence in the treatment’s effectiveness and noted improvements in self-esteem (Contribution 10).

## 5. Technological Advances and Assessment Methods

The use of complementary diagnostic technologies in clinical practice, such as optical coherence tomography (OCT), has transformed how we assess myopia progression [13,14].

Breher et al. compared peripheral refraction profiles with OCT and concluded that this technique provides a more accurate estimate of retinal eccentricity, especially in eyes with high myopia. These results suggest that OCT-based methods may be preferred in future studies to monitor myopia progression more reliably (Contribution 11).

The integration of machine learning is also emerging as a tool for analyzing large volumes of clinical data. Wu et al. applied machine learning models to evaluate how 19 different variables influence intraocular pressure in myopic children treated with atropine. The eXtreme gradient boosting (XGBoost) model was identified as the most effective for predicting variations in intraocular pressure, highlighting the importance of advanced predictive methods in myopia management (Contribution 12).

## 6. Combined Therapies: A Promising Pathway

The combination of treatments has shown significant promise. Research indicates that this synergistic approach not only slows axial elongation but also enhances visual acuity in children with myopia [15].

Sánchez-González et al. reviewed studies that combined orthokeratology with low-dose atropine and concluded that this synergistic approach is more effective than orthokeratology alone. This reinforces the notion that personalized and combined interventions may represent the future of myopia management (Contribution 13).

## 7. Challenges and Future Perspectives

Despite significant advancements, many challenges remain. Studies have highlighted the need for further long-term investigations to assess the sustainability of interventions [16,17,18,19].

Varnas et al. demonstrated that the efficacy of multifocal lenses may diminish after the first year of use, suggesting the necessity for complementary approaches to maintain long-term effectiveness (Contribution 14).

Furthermore, genetic variability and individual responses to treatments, as evidenced by the genetic studies of Alvarez-Peregrina et al. (Contribution 3), indicate that a more personalized approach may be required. Personalization could include not only the choice of treatment but also adjustments in dosage and method based on genetic profiles and environmental characteristics.

In addition to these interventions, Queirós et al. proposed a new mathematical model to estimate axial length (AL), essential for assessing myopia progression and monitoring treatment effects. This model was tested in cross-sectional and longitudinal samples and proved effective in estimating changes in axial growth among youths aged 9 to 24 years, even with some discrepancies relative to actual values. This approach enables practitioners without access to AL measurement equipment to monitor myopia progression more practically and economically (Contribution 15).

## 8. Conclusions

This Special Issue offers a valuable contribution to the scientific literature on the control of myopia progression in children. Advances in optical and pharmacological interventions, along with new technological approaches and a deeper understanding of genetic factors, are shaping a promising future for myopia management. However, ongoing investigation is necessary, particularly in combined therapies and long-term studies, to ensure treatments are sustainable and accessible. The future of myopia control appears to lie in the combination of personalized strategies, grounded in both clinical evidence and individual factors, that maximize efficacy while minimizing risks for future generations.
